# Powders in Papers

**Published:** 1890-07-12

**Authors:** 


### POWDERS IN PAPERS.

Dr. Arthur Hoffmann, of Darmstadt, recommends the administration of powders in the Japanese paper marked "Usego," which is imported in sheets 50 cms. by 36 cms., each sheet weighing less than 30 grs. It is of a yellowish-white tint, a silky softness, and, though so transparent that printing can be easily read through it, surprisingly tough. The powder is to be placed on a small square, the corners of which are brought together, strongly twisted, and the "cord" thus formed cut short with scissors. The packet is swallowed as easily as a pill, and, as may be seen by dropping one in water, unwinds and discharges its contents when it has reached the stomach.

